# Detection of sweet corn seed viability based on hyperspectral imaging combined with firefly algorithm optimized deep learning

**DOI:** 10.3389/fpls.2024.1361309

**Published:** 2024-05-01

**Authors:** Yi Wang, Shuran Song

**Affiliations:** ^1^ College of Electronic Engineering (College of Artificial Intelligence), South China Agricultural University, Guangzhou, China; ^2^ College of Software Engineering, Guangdong University of Science and Technology, Dongguan, China

**Keywords:** sweet corn seeds, seed vitality, deep learning, spectral image, firefly algorithm

## Abstract

The identification of sweet corn seed vitality is an essential criterion for selecting high-quality varieties. In this research, a combination of hyperspectral imaging technique and diverse deep learning algorithms has been utilized to identify different vitality grades of sweet corn seeds. First, the hyperspectral data of 496 seeds, including four viability-grade seeds, are extracted and preprocessed. Then, support vector machine (SVM) and extreme learning machine (ELM) are used to construct the classification models. Finally, the one-dimensional convolutional neural networks (1DCNN), one-dimensional long short-term memory (1DLSTM), the CNN combined with the LSTM (CNN-LSTM), and the proposed firefly algorithm (FA) optimized CNN-LSTM (FA-CNN-LSTM) are utilized to distinguish spectral images of sweet corn seeds viability grade. The findings from the experimental analysis indicate that the deep learning models exhibit a significant advantage over traditional machine learning approaches in the discrimination of seed vitality levels, boasting a classification accuracy exceeding 94.26% in test datasets and achieving an accuracy improvement of at least 3% compared to the best-performing machine learning model. Moreover, the performance of the FA-CNN-LSTM model proposed in this study demonstrated a slight superiority over the other three models. Besides, the FA-CNN-LSTM achieved a classification accuracy of 97.23%, representing a significant improvement of 2.97% compared to the lowest-performing CNN and a 1.49% enhancement over the CNN-LSTM. In summary, this study reveals the potential of integrating deep learning with hyperspectral imaging as a promising alternative for discriminating sweet corn seed vitality grade, showcasing its value in agricultural research and cultivar breeding.

## 1 Introduction

Corn, as a primary source of food and fuel, is widely cultivated worldwide (Erenstein et al., 2022). Sweet corn seeds have become increasingly popular as a new genetically improved variety due to their delicious taste and exceptionally high nutritional value. Due to being genetically modified varieties, their seed germination rate is not very high (Wang B. et al., 2022). However, seed vitality is a crucial factor in determining seed germination rate. Therefore, identifying the vitality of sweet corn seeds is very meaningful (Wang Z. et al., 2022).

Traditional seed vitality testing methods such as germination and tetrameter tests can visually distinguish seed vitality levels but come with drawbacks, including long experimental periods, high costs, and significant damage to the seeds. Nevertheless, various innovative physical testing techniques, such as X-ray analysis, nuclear magnetic resonance spectroscopy, Fourier spectroscopy, and Raman spectroscopy, have also been employed to a certain degree. However, they face challenges including low efficiency, complex operation, and limitations in batch testing (Musaev et al., 2021). Hyperspectral imaging technology, a rapid and non-invasive assessment tool, has found extensive application in evaluating the quality and characteristics of fruits, vegetables, and crops (Khan et al., 2022). Compared to conventional RGB image detection methods, hyperspectral imaging technology proficiently scrutinizes the intrinsic composition and surface texture attributes of the target under examination, which has been proved in tomato ripeness detection, analysis of pesticide residues on tea leaves’ surfaces, and assessment of the extent of physical mold contamination, among other aspects. Furthermore, this technology has been used to detect the moisture content, nutritional components, and diseases in sweet corn seeds. However, there is currently limited research on the identification of sweet corn seed vitality grades using the hyperspectral technique. Hyperspectral imaging systems can capture spectral data by measuring the reflected light at various wavelengths from the surfaces of objects, concurrently collecting relevant image information, enabling precise detection and discrimination of the objects being tested. Hence, the application of hyperspectral imaging for assessing the vitality levels of sweet corn seeds represents a viable and practicable approach (Lu et al., 2020).

Integrating conventional machine learning algorithms with hyperspectral imaging technology is a widely adopted nondestructive detection method. Wang et al. (2018) introduced the utilization of near-infrared hyperspectral imaging technology as a reasonable and precise method for identifying haploid maize kernels. In their study, two representative maize varieties were used as the experimental object, and the spectral features of hyperspectral imaging were utilized to investigate the impact of embryo orientation on the haploid identification model. The results showed that the correct acceptance rates for both haploid and diploid test sets were 99%, with error acceptance rates below 1%, indicating a high level of accuracy. Yang et al. (2023) proposed a corn seed variety identification method that relies on random subspace ensemble learning in conjunction with hyperspectral imaging technology. Firstly, spectral information from the maize endosperm region was collected, and several preprocessing methods were applied to analyze the spectral data. Finally, a corn seed classification detection model was constructed using MSC-IRIV-RSEL. The experimental findings demonstrated that the suggested approach attained a classification accuracy of 95.56%. Liu et al. (2020) presented the application of near-infrared (930-2500nm) hyperspectral imaging technology for the assessment of starch content in individual corn seeds. The PLSR and LMA were employed to predict the starch content. The outcomes revealed that LMA demonstrated a correlation coefficient of 0.96 and a root mean square error 0.98 on the prediction dataset. Zhang et al. (2022) have devised an incremental learning model to discriminate corn seed varieties. They collected hyperspectral image data from five different maize varieties, conducted preprocessing, and applied various classification models. The relevant conclusions indicated that their proposed method outperformed other classification algorithms, achieving an accuracy rate close to 100%. Wang et al. (2021)conducted a classification study on the maturity of corn seeds using Near-Infrared Hyperspectral Imaging (NIR-HSI) technology. Initially, hyperspectral images of corn seeds, obtained from both the embryo and endosperm sides, were collected within the spectral range of 1000 to 2300 nanometers. Subsequently, the classification model was constructed using PLS-DA, DT, and AdaBoost. The results showed that the PLS-DA algorithm achieved classification accuracies of 98.7%. As a result, the amalgamation of conventional machine-learning algorithms with spectral information has yielded favorable outcomes in identifying corn varieties and other related factors.

Due to the greater economic significance of sweet corn seeds, their vitality has garnered growing and extensive attention. However, the classification and identification of the vitality of sweet corn seeds using hyperspectral imaging technology were rarely reported. Apart from the commonly employed machine learning algorithms, several renowned deep learning models have been devised to analyze hyperspectral data (Yao et al., 2023a). These models are recognized for their notable advantages in extracting profound insights from images (Saha and Manickavasagan, 2021). A series of deep learning models have been put forth across various domains, including Recurrent Neural Networks (RNNs), Long Short-Term Memory (LSTM), Convolutional Neural Networks (CNN), and others. Some one-dimensional deep learning models have already found application in seed vitality detection have already found application in seed vitality detection (Li et al., 2019). Zhang et al. (2021) introduced an innovative approach by integrating deep learning techniques with the near-infrared hyperspectral technique (874-1734nm) to distinguish between different coated corn seeds. The spectral reflection values were obtained from this imaging method as the primary input for training one-dimensional CNN and LSTM models. The outcomes of their experiments revealed remarkable results, with all models consistently achieving a classification accuracy surpassing 90%. Bi et al. (2022) introduced an innovative approach known as the swim transformer to enhance the recognition of corn seeds. Their model integrated feature attention mechanisms and multi-scale feature extraction techniques, substantially enhancing its performance. The numerical analysis of the results demonstrated excellent classification performance on both the test and training datasets. Xu et al. (2022) have introduced a rapid, nondestructive, and efficient approach for the detection of defects in corn seeds by integrating hyperspectral imaging (HSI) technology with deep learning. Initially, they formulated a Convolutional Neural Network structure (CNN-FES) based on a feature selection mechanism. Furthermore, they developed a Convolutional Neural Network architecture (CNN-ATM) incorporating an attention-based classification mechanism for classifying one-dimensional spectral data. The results indicate that the designed CNN-ATM performs similarly to the three aforementioned methods across the entire wavelength spectrum. It achieves classification accuracy of over 90% on both the training and test datasets. Yao et al. (2022) proposed an iterative semi-supervised CNNs framework by means of active learning and superpixel segmentation techniques, dubbed semi-active CNNs (SA-CNNs) for HSI classification. Firstly, a CNN-based on a small-scale unbiased labeled set was used. Then, the reliable samples consist of two parts: high label homogeneity and most informativeness were actively selected from superpixel segments. The experimental results show substantial performance improvements of the proposed SA-CNNs over other similar competitors. Zhang et al. (2020) proposed hyperspectral imaging combined with the DCNN to identify corn seed varieties. Three models, namely DCNN, KNN, and SVM, were employed to construct the classification models. The findings revealed that the DCNN model achieved a training accuracy rate of 100%. Yao et al. (2023b) proposed a novel multimodal deep learning framework by extending conventional ViT with minimal modifications (ExViT). First, a multimodal RS image patch with parallel branches of position-shared ViTs extended with separable convolution modules was used. Then, a cross-modality attention (CMA) module was employed in RS scenes by exploiting pixel-level spatial correlation. The experimental results effectively demonstrate the effectiveness of the proposed method. Fan et al. (2023) employed hyperspectral imaging technology in combination with a multi-scale three-dimensional Convolutional Neural Network (3DCNN) to discern the vitality of individual seeds. Utilizing a voting algorithm to amalgamate the outcomes from all small blocks associated with the same seed, they determined an individual seed’s viability. The results indicated that the multi-scale 3DCNN model achieved a discrimination accuracy of 90.67% when assessing the vitality of individual seeds in the test dataset.

These studies collectively demonstrate that spectral images acquired from hyperspectral imaging systems can be employed to train deep learning models for distinguishing various levels of seed vitality. However, the current input data for deep learning algorithms mostly consists of one-dimensional spectral information without integrating spectral information with image data for training neural networks. Additionally, the temporal correlation of spectral data acquisition is often overlooked. Therefore, optimizing neural network models through combination is advantageous for better training on hyperspectral images, leading to the extraction of more effective features and ultimately achieving efficient detection of sweet corn seed vitality.

The aim of this study is to utilize a range of deep convolutional algorithms and their enhanced network models to achieve a rapid nondestructive assessment of the vitality of sweet corn seeds. The specific objectives are outlined as follows:

(1) To evaluate the effectiveness of a CNN-LSTM model optimized with the firefly algorithm for the detection of sweet corn seed vitality.
(2) To employ various deep learning models to develop classification models for sweet corn seed vitality using spectral images.
(3) To assess the viability of standard machine learning algorithms for the identification of sweet corn seed vitality.

## 2 Materials and methods

### 2.1 Sample preparation

In this study, a total of 600 Yuetian 29 sweet corn seeds were prepared, from which 496 seeds exhibiting complete grains and uniform size were carefully chosen. These seeds were acquired from the Guangdong Academy of Agricultural Sciences’ Tmall shopping platform and were harvested in April 2023. All samples were divided into four groups, each group containing 124 samples, which were packaged as purchased and stored at a consistent temperature of 4°C until the commencement of the experiment. Prior to the experiment, the 496 chosen sweet corn seeds were positioned inside an artificial aging chamber with a temperature set at 50°C and a relative humidity level of 25%. The seeds were placed in the aging chamber for 6 hours, 12 hours, and 24 hours, respectively, and were subsequently removed and labeled as B, C, and D grades. Besides, the samples that have not been aged are labeled A. After aging, all the seeds were dried in a 20°C incubator to regain their original weight.

### 2.2 Hyperspectral information acquisition

**Figure 1:**
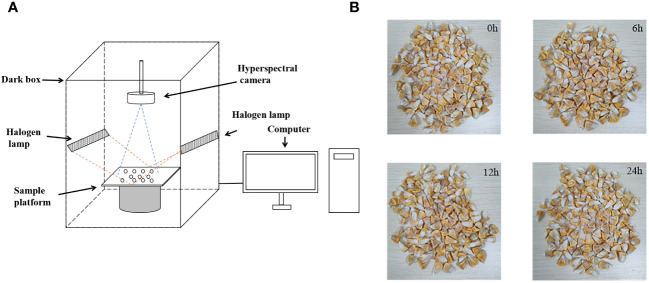
Hyperspectral data collection. **(A)** hyperspectral imaging system; **(B)** artificially aged seeds.

Hyperspectral data was collected using the GaiaField-V10E hyperspectral imaging system during the experiment. This equipment was manufactured by Jiangsu Dualix Spectral Imaging Technology Co., Ltd, located in Wuxi, Jiangsu, China. The system primarily consists of components such as a hyperspectral camera, a spectral dark box, a halogen lamp, a computer, and a sample platform. This state-of-the-art apparatus boasts a broad spectral range spanning from 388nm to 1025nm, harnessing a comprehensive collection of 360 bands designed to capture intricate spectral data. The equipment and artificially aged seed samples are shown in **Figure 1**.

The ideal distance between the camera lens and the sample was meticulously set at 68 cm to ensure the capture of clear and precise images. Furthermore, the exposure time and camera scan speed were configured at 12 ms and 0.6 mm/s, respectively. Besides, the sweet corn seeds with different aging grades were numbered to carry out better germination experiments and analyze seed vitality. After these operations, spectral images of the samples were expertly acquired. Finally, the image correction for black and white plate data was performed using the following Equation 1: (Tang et al. 2023)


(1) $$I=\frac{I_{\text{org}}-I_{\text{dark}}}{I_{\text{white}}-I_{\text{dark}}}$$

where *I_org_, I_dark_, I_white_*, and *I* represent the original hyperspectral image, black reference, white reference, and calibrated image, respectively. Subsequently, the calibrated image was utilized to extract the local average spectrum or directly serve as the convolutional neural network input.

### 2.3 Standard germination test

Following the acquisition of hyperspectral data from seeds of four different grades, a germination test was conducted in accordance with the standards of the International Seed Testing Association (ISTA) to assess the impact of artificial aging on sweet corn seeds (Pang et al., 2021). The seeds were placed on a germinating tray with germinating paper according to the number, and all the germinating samples were placed at a room temperature of about 25-30°C for the germinating experiment. The germination process lasts seven days, during which a certain amount of water is applied according to the moisture level of the germinating paper. On the seventh day of germination testing, measure the length of each seedling. According to the ISTA standards, seeds with embryonic shoot lengths exceeding 1cm are considered germinated or viable. Therefore, on the seventh day of the experiment, tally the number of germinated seeds based on shoot length and calculate the germination rate of the seeds. The germination rate results for the seeds are presented in **Table 1**. The findings indicate that, as aging time increased, the germination percentage of the seeds decreased. The germination of aged seeds significantly differed from that of normal seeds. It is evident that creating sweet corn seeds with varying levels of viability through artificial aging is achievable. Still, producing robust seedlings under favorable conditions is challenging for these treated seeds.

**Table 1:** Statistical results of different germination numbers.

Statistical object	A (0h)	B (6h)	C (12h)	D (24h)	Total
Total number of samples	124	124	124	124	496
Number of germinated seeds	113	98	87	74	372
Germination percentage	91.1%	79.0%	70.2%	59.7%	75.0%

To further differentiate seeds of varying vigor, categorize seeds of each aging level into four vigor grades based on shoot length: high vigor (greater than 7cm), medium vigor (3-7cm), low vigor(1-3cm), and no vigor(less than 1cm). For the sake of simplicity, we use abbreviations to represent different vigor levels: High Vigor (HV), Medium Vigor (MV), Low Vigor (LV), and No Vigor (NV), respectively. The statistical results of different germination lengths are shown in **Table 2**. It can also be seen from **Table 2** that seed vitality decreases to some extent with the degree of aging. Finally, the numbers of high, medium, low, and non-viable seeds contained in the seed collection of Guangdong Sweet 29 were 125, 120, 127, and 124, respectively, obtained after germination experiments. According to the number of seeds, the corresponding spectral information was extracted for the subsequent seed vigor identification study.

**Table 2:** Statistical results of different germination lengths.

Statistical object	A (0h)	B (6h)	C (12h)	D (24h)	Total
high vigor (>7cm)	43	36	29	17	125
medium vigor (3-7cm)	45	32	22	21	120
low vigor (1-3cm)	25	30	36	36	127
no vigor (<1cm)	11	26	37	50	124

### 2.4 Spectral extraction and preprocessing

**Figure 2:**
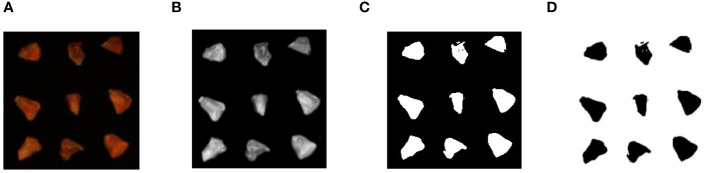
Spectral data extraction process. **(A)** 635.77nm wavelength image; **(B)** image processing; **(C)** image segmentation; **(D)** ROI of every corn seed.

In the extraction of hyperspectral data, the average reflection spectral value of the region of interest in the spectral image is calculated after correcting the hyperspectral images. This experiment obtained the average reflectance spectral values corresponding to the whole corn seed region. The image data format generated by the hyperspectral imaging system is represented as *m*×*n*×*w*, where *m* stands for image length, *n* for image width, and *w* for the number of spectral bands. In the case of the GaiaField-V10E hyperspectral imaging system, the spectral image data matrix is 1211×960×360. There were four distinct types of sweet corn seeds with varying vitality levels, and each image contained 50 seeds. Consequently, a total of 12 images were acquired. The spectral data of 496 sweet corn seeds were selected through a germination experiment. Then, image processing technology automatically extracts each sample’s average reflection spectral value in each band. The entire automated process for extracting Regions of Interest (ROI) from the calibrated spectral image segment is illustrated in **Figure 2**. Step 1: Initially, hyperspectral images were read, and spectral data were analyzed. The reflectance image of sweet corn seeds at 635.77nm was chosen in the first step because it exhibited clear outlines and distinct appearances. Step 2: The captured grayscale image underwent filtering and enhancement operations before image segmentation. Step 3: The Otsu algorithm was utilized to derive the binary image. Step 4: Subsequently, the binary image served as the mask image. In the next step, the mask image and calibrated images were used to extract complete sweet corn seed images. Step 5: Finally, the spectra of all pixels within the ROI were averaged at each wavelength for each seed.

### 2.5 Different classification models

#### 2.5.1 Classic machine learning algorithms

The Support Vector Machine (SVM) is a robust supervised classification method extensively used for addressing nonlinear classification, function estimation, and pattern recognition tasks (Luan and Tsai, 2021). Its fundamental principle revolves around establishing a hyperplane capable of effectively segregating the training dataset within a high-dimensional feature space, maximizing the geometric margin. This study selected the Radial Basis Function (RBF) as the kernel function because of its effectiveness in dealing with nonlinear data. Achieving a well-fitted model requires carefully determining two critical parameters: the penalty coefficient (*c*) and the kernel parameter (*g*). The grid-search technique has been utilized to identify the optimal values for *c* and *g*. The search range for these parameters was set to 2^-8^-2^8^. The Extreme Learning Machine (ELM) is a formidable machine learning algorithm employed in classification and regression tasks. ELM stands out for its remarkable efficiency, particularly in solving nonlinear problems. The core concept behind ELM is to randomly initialize the weights and biases of the hidden layer and then focus on training only the output layer. To obtain an effective ELM model, it’s crucial to tune two key parameters: the number of hidden neurons (*N*) and the activation function. The weight parameters connecting neurons within the ELM structure were stochastically generated and remained entirely unrelated to the training data (Bansal et al., 2022).

#### 2.5.2 Convolutional neural networks

The information collected from sweet corn seeds of different vitality levels by the hyperspectral imaging system includes spectral and image data. Therefore, some typical neural network models utilize spectral images and data to construct seed vitality identification models.

Convolutional Neural Networks (CNNs) are a class of deep learning models specifically tailored for analyzing and processing data with grid-like structures, particularly images and videos. CNNs are founded on the fundamental concept of hierarchical feature extraction through convolutional and pooling operations, enabling them to excel in various computer vision tasks, such as image recognition, classification, and segmentation. A typical CNN architecture consists of multiple layers, including convolutional layers, pooling layers, and fully connected layers, all working together to automatically learn and extract high-level features from input data. This process greatly aids in comprehending complex patterns and structures (Li et al., 2021).

**Figure 3:**
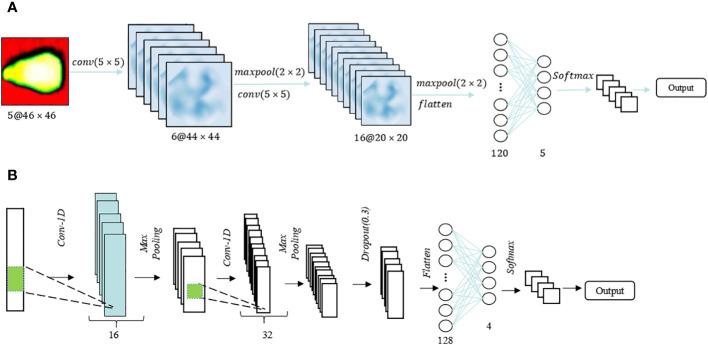
CNN models. **(A)** CNN for image; **(B)** 1DCNN for spectrum.

Drawing from sweet corn seeds’ spectral and image characteristics at varying vitality levels, this study introduces dedicated 2DCNN and 1DCNN models designed for vitality detection. The structural diagrams of these models are presented in **Figures 3A, B** for the two-dimensional and one-dimensional CNNs, respectively. The 2DCNN comprised two convolutional layers with a kernel size of 5×5 each, and the number of filters was configured as 6 and 16, respectively. The size of the maximum pooling layer was set at 2×2. Before training the CNN model using spectral images, each seed image was cropped to a size of 46×46. In addition, for data dimensionality reduction, Principal Component Analysis (PCA) is applied to transform the original 46×46×360 spectral image data into a reduced format of 46×46×5. Consequently, this reduced image is used as the input for a fully connected layer consisting of 120 neurons, followed by another layer with 5 neurons, both of which employ the Rectified Linear Unit (ReLU) activation function. The entire model is trained on the samples using the stochastic gradient descent (SGD) optimization technique. In contrast to the 2DCNN, the 1DCNN transforms data into a 1D format by reducing its dimensionality. Similarly, the 1DCNN also comprises two convolutional layers with a size of 1×3, and deep feature down-sampling is achieved through maximum pooling with a size of 1×2. The number of neurons in the fully connected layer is reduced from 128 to 4, and ultimately, softmax is employed for viability classification. To mitigate the risk of overfitting and introduce regularization, a dropout layer with a rate of 0.2 is incorporated before each fully connected layer. The activation function settings remain consistent with those of the 2DCNN.

#### 2.5.3 Long short-term memory

**Figure 4:**
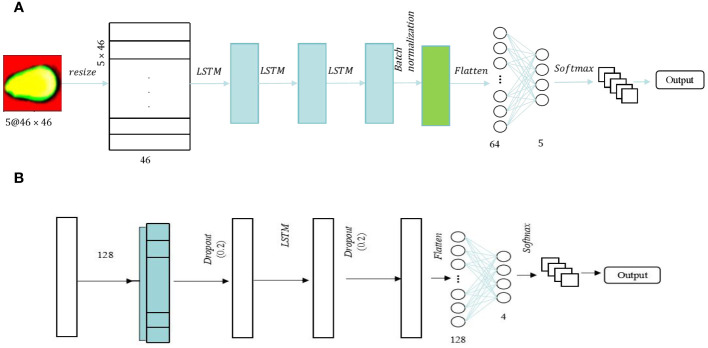
LSTM models. **(A)** LSTM for the image; **(B)** 1DLSTM for the spectrum.

Long Short-Term Memory (LSTM) is a distinctive variant of recurrent neural network (RNN) architecture that has garnered substantial recognition in the realm of deep learning [23]. LSTM is engineered to address the challenge of capturing and modeling long-range dependencies within sequential data, which is crucial for various complex tasks such as time series analysis and natural language processing. This intrinsic capability makes LSTM a formidable choice for diverse applications that demand the modeling and prediction of sequential data with intricate temporal dependencies. Two-dimensional (2D) and One-dimensional (1D) LSTM frameworks are employed to process spectral and image features. The optimal architectures for these frameworks are depicted in **Figures 4A, B**, respectively. The Spectral-LSTM model consisted of an LSTM block and two fully connected layers, and a dropout layer with a value of 0.2 was added in front of these two parts to prevent overfitting. The difference is that in the image-LSTM model, batch normalization was added between the two parts in addition to three LSTM blocks and two fully connected layers. Before softmax classification, exponential linear unit (ELU) was used as the activation function in the image-LSTM model. This method tried to close the average value of the output of the activation function to zero, thereby speeding up the learning speed and avoiding the problem of the disappearance of the gradient through the identification of positive values.

#### 2.5.4 CNN-LSTM model

**Figure 5:**
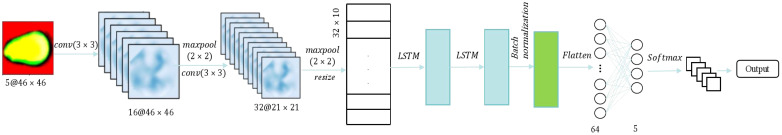
CNN-LSTM for image.

Using PCA for dimensionality reduction before training a CNN network can reduce the amount of training data required to some extent. However, it also results in the loss of some valuable spectral information. LSTM networks are effective models for handling time series data, and the spectroscopic data collected in the experiment has a certain temporal sequence. In this study, we propose a hybrid architecture known as CNN-LSTM. The CNN-LSTM model is designed to integrate spectral fusion features into the network architecture, ultimately enhancing its performance. **Figure 5** illustrates the CNN-LSTM framework for evaluating the viability of sweet corn seeds. Two convolutional layers with a 3×3 kernel size were employed to extract features from spectral images. The output image following the CNN convolutional processing served as input for two LSTM blocks and batch normalization was added. Subsequently, two fully connected layers, comprising 128 and 5 neurons, were incorporated, with the activation function remaining as the Exponential Linear Unit (ELU).

Deep learning models typically have numerous hyperparameters, many of which are determined based on personal experience or multiple trial-and-error attempts. In the proposed CNN-LSTM model, two crucial parameters are batch size (bs) and learning rate (lr). The lr determines the speed of model learning, with a higher lr resulting in faster training but potentially leading to a significant decrease in network performance. Small batch sizes are typically employed during network training. Each epoch subdivides the training samples into several groups, known as the batch size, and feeds them into the network for training. In general, a larger batch size leads to a more accurate determination of the descent direction.

#### 2.5.5 The CNN-LSTM optimized by the Firefly algorithm

To further boost the performance of the CNN-LSTM model, we suggest employing the Firefly algorithm (FA) to optimize two crucial hyperparameters within the model: learning rate (lr) and batch size (bs). The Firefly Algorithm is a population-based stochastic search algorithm. Each firefly is randomly positioned within the search space of the objective function. The brightness of each firefly is tied to the fitness value of the objective function at its current location. Brighter fireflies represent positions that yield superior values for the objective function. Each firefly is attracted to brighter fireflies, which motivates them to move in search of better solutions. As the population evolves, the algorithm ultimately converges to effective solutions for optimization problems. The algorithm is detailed and explained as Equations 2–5 (Kumar and Kumar, 2021).

For a *D*-dimensional search space and a total population size of *NP* in the population, the position of the *i*-th firefly (*i* = 1, 2,…, *j*, *NP*) is represented as *X_i_* =(*x_i_*_1_, *x_i_*_2_,…,*x_i_*_D_).

In nature, fireflies rely on the intensity of their own light to attract potential mates or companions. The attraction *β*(*r*) between two fireflies is primarily determined by their luminous intensity, which can be expressed using the formula (2).


(2) $$\beta \left(r\right)=\beta_{0}\times e^{-\gamma \times r_{i,j}2}$$

where *γ* is a fixed light absorption coefficient, *β*_0_ is the attractiveness at *r* = 0, the distance *r_i,j_* between any two fireflies *i* and *j* at *X_i_* and *X_j_* can be computed according to Euclidian distance:


(3) $$r_{i,j}=\left\|X_{i}-X_{j}\right\|=\sqrt{\sum_{d=1}^{D}{\left(x_{i,d}-x_{j,d}\right)}^{2}}$$

For fireflies *x_i_* and *x_j_*, if the brightness of firefly *x_j_* is greater than that of firefly *x_i_*, firefly *x_i_* will move toward firefly *x_j_*. The position update formula is as follows:


(4) $$x_{i}^{t+1}=x_{i}^{t}+\beta_{0}e^{-\gamma r_{i,j}^{2}}\left(x_{j}^{t}-x_{i}^{t}\right)+\alpha \left(rand-\frac{1}{2}\right)$$

where *α* is called the step factor, *rand* represents uniformly distributed random number within [0, 1].

**Figure 6:**
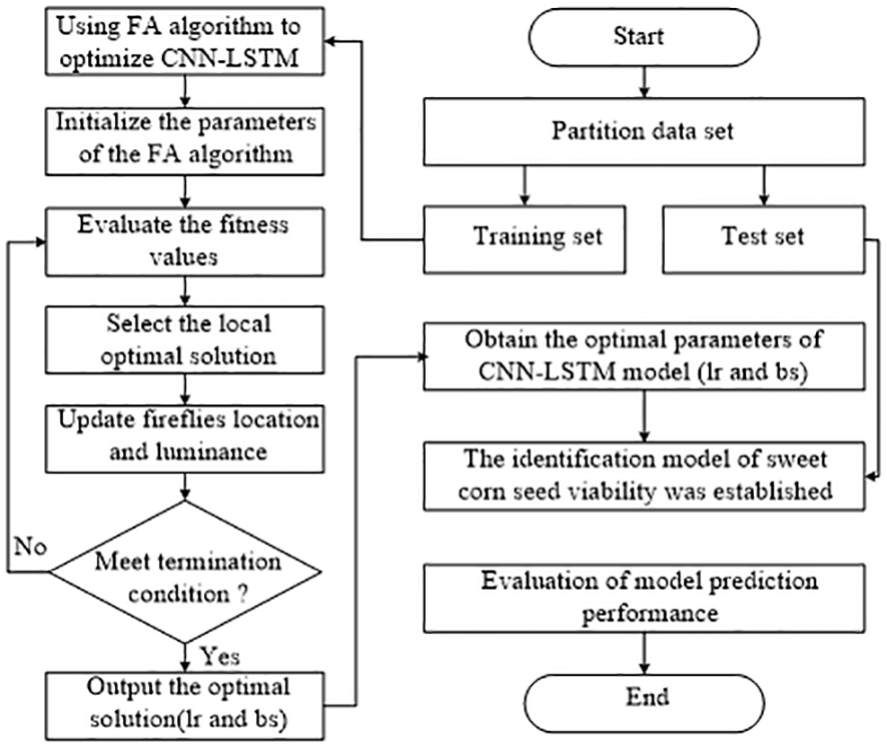
The structure of FA optimized the CNN-LSTM.


Therefore, the Firefly Algorithm flowchart for optimizing the CNN-LSTM model is depicted in **Figure 6**. Specifically, the objective function for optimizing the CNN-LSTM model with the FA algorithm is defined as follows:

(5) $$f_{obj}=1-\frac{sum\left(T_{r}=T_{v}\right)}{T_{r}}$$

where, *T_r_* represents the actual number of sample labels, and *T_s_* represents the predicted sample labels. Therefore, the smaller the value of the objective function, the more reliable the model performance, and the optimized parameters are also the best. The specific description of the whole algorithm framework is explained in the following steps: Step 1: Partitioning the sample dataset. Step 2: The parameters of the CNN-LSTM model are optimized by using the FA algorithm in the training set. Step 3: Initialize the parameters of the FA algorithm. Step 4: Evaluate the fitness function, select the optimal individual and local optimal solution, and update the population information. Step 5: Determine whether the algorithm meets the termination condition. Step 6: Output the optimal solution(lr and bs). Step 7: The CNN-LSTM model is constructed with the optimal parameters, and the performance of the model is evaluated on the test set.

## 3 Experimental results and analysis

### 3.1 Spectral characteristics analysis

**Figure 7:**
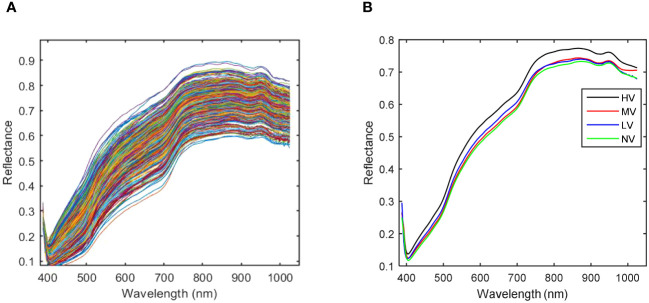
The spectra curves of sweet corn seeds with different vigor. **(A)** Full spectra curves; **(B)** Average spectra curves.

**Figure 7** shows the spectra curves of all samples and the average spectra of four kinds of sweet corn seeds with different vigor, respectively. The spectral range of 388-1025 nm was employed to study the spectral characteristics of sweet corn seeds. While the spectral curves of four different grades of vitality seeds exhibited a fundamental similarity, distinctions arose in terms of the specific wavelengths of spectral absorption peaks and the corresponding reflectance values at each band. As evident from the averaged spectral curve depicted in **Figure 7B**, variations exist in the average spectral curves corresponding to varying degrees of aging. Notably, the spectral reflection values tend to decrease with prolonged aging duration. Among these observations, the distinct dissimilarity between the spectral curve of HV (no aging) and that of the other three aging categories is noteworthy. The spectral curves of the other three kinds of sweet corn seeds with different aging degrees are difficult to distinguish. The value of the average spectra of LV is the highest compared to NV and MV at wavelengths of 400-750nm. Moreover, the average spectral value of MV is the highest compared with LV and NV in the 750-1025nm range. The average spectral curves of NV and LV are very similar to each other to some extent. Broadly speaking, the spectral curves of the four distinct types of sweet corn seeds, each characterized by varying levels of vigor, exhibit notable distinctions. As a result, the extraction of spectral attributes for the purpose of variety classification appears to be a viable approach.

**Figure 8:**
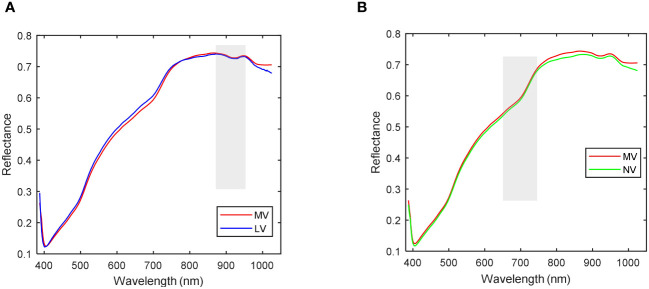
Correlation analysis of spectra. **(A)** Average spectrum with correlation analysis of MV and LV; **(B)** Average spectrum with correlation analysis of MV and NV.

As depicted in **Figure 7B**, it becomes evident that the spectral curves of the HV seeds stand out from those of the other three vitality levels. Consequently, MV, LV, and NV are considered for correlation analysis. The discrepancies in average spectra between MV, LV, and NV are illustrated in **Figure 8**. The shaded gray areas in **Figure 8** indicate that the wavelengths corresponding to the three vitality levels do not exhibit significant differences (*p*< 0.01). Furthermore, MV and LV exhibit substantial differences except in the 870-930nm range, and MV and NV show significant differences except in the 650-760nm range. The findings from **Figure 7** and **Figure 8** collectively suggest that all four vitality levels can be effectively visually distinguished based on the average spectral profiles of their different vitality levels. Consequently, the training of machine learning models can effectively accomplish the classification of these different vitality levels.

### 3.2 Comparison of the effects of traditional models under different preprocessing strategies

**Figure 9:**
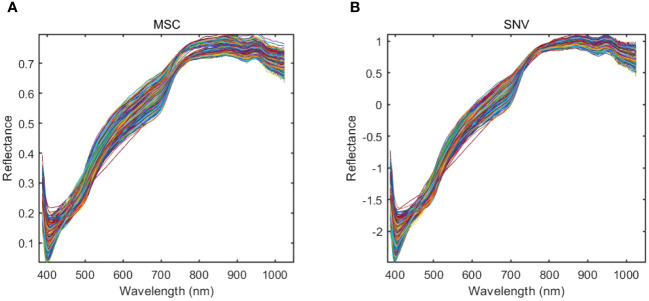
The preprocessing results of hyperspectral data. **(A)** MSC; **(B)** SNV.

During the process of collecting sample data in a spectral imaging system, it is difficult to avoid being disturbed by various types of noise information. Translation: Therefore, preprocessing of sample data can effectively enhance the accuracy of the data. Standard Normative Variables (SNV) and Multiple Scattering Correction (MSC), as two primary data preprocessing methods, can effectively remove peaks and spikes from the data while preserving the primary characteristics of the samples, resulting in smoother data. The preprocessing results of hyperspectral data are shown in **Figure 9**. The MSC can effectively enhance the correlation between spectral data, reduce spectral variations caused by different scattering levels, and thereby effectively reduce the impact of noise on data analysis, as shown in **Figure 9A**. On the other hand, SNV can effectively standardize and normalize spectral data, thereby improving spectral resolution, as shown in **Figure 9B**. After data preprocessing, the spectral data become smoother.

This paper used SVM, ELM, 1DLSTM, and 1DCNN to establish the models for determining the viability of sweet corn seeds under a hyperspectral full band. All the sample data were randomly divided into a training set and a test set according to 7:3. The experimental results of traditional models using different preprocessing strategies are shown in **Table 3**. As demonstrated by the findings in **Table 3**, the classification results for the original spectral data indicate that 1DCNN outperformed other methods, achieving an impressive accuracy of 91.08%. This accuracy is notably 4% higher than that of SVM, which exhibited the poorest performance among the tested algorithms. Moreover, the utilization of preprocessed data notably enhances the model’s classification accuracy compared to using the original spectral data. Among the various preprocessing methods evaluated, data subjected to the MSC preprocessing yielded the most impressive classification results, boasting the highest accuracy at 92.03%. This performance surpassed that achieved by the SNV preprocessing across all four models. In terms of model performance, 1DCNN has the best performance, followed by ELM, 1DLSTM is inferior to ELM, and SVM has the worst performance.

**Table 3:** Classification accuracy of traditional models using different preprocessing strategies.

Model	Preprocessing method					
	Original spectra		MSC		SNV	
	Training accuracy (%)	Test accuracy (%)	Training accuracy (%)	Test accuracy (%)	Training accuracy (%)	Test accuracy (%)
SVM	92.35	87.16	92.45	**89.21**	92.31	87.55
ELM	93.68	90.57	94.03	**91.23**	93.02	90.69
1DLSTM	91.29	89.33	92.35	**90.52**	91.60	89.87
1DCNN	93.45	91.08	93.59	**92.03**	93.21	91.54

The bold font indicates that the algorithm has the best results compared to the other parties.

**Figure 10:**
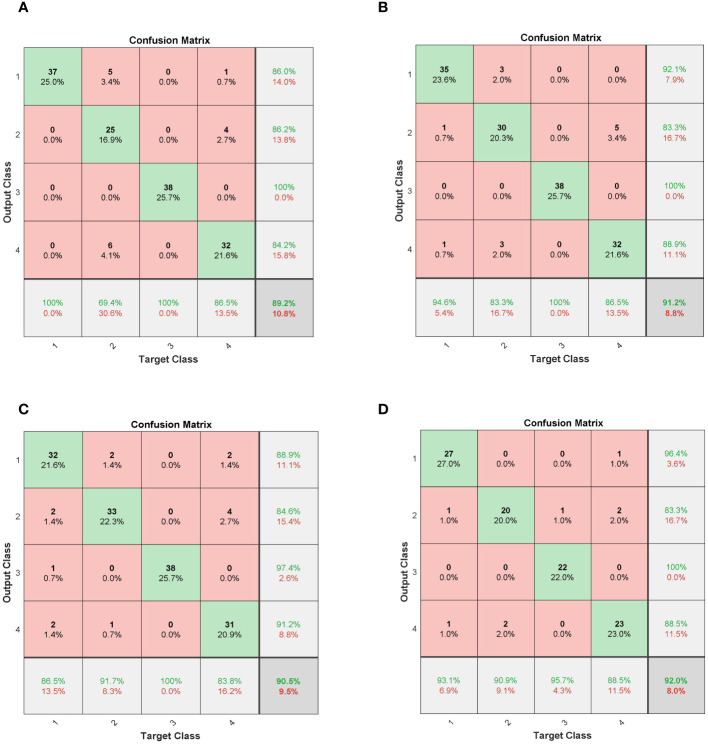
Confusion matrices for different models. **(A)** SVM; **(B)** ELM; **(C)** 1DLSTM; **(D)** 1DCNN.

To better analyze the model’s performance in identifying seeds with different vigor levels, the corresponding confusion matrices of the above models are shown in **Figure 10**. As can be seen from the analysis in **Figure 10**, all models were significantly easier to distinguish between high vitality and low vitality samples, which is basically consistent with the analysis results of the average spectral curves shown in **Figure 7**. For the deep learning models, its models are built through continuous training and learning, which has a relatively average classification accuracy rate for each type of sample. On the whole, the classification error rate of the model is the highest for the moderately active and inactive samples, which is basically consistent with the correlation analysis results in **Figure 8**. Of course, the result of this reason is that the characteristics of the samples are very similar.

### 3.3 Comparison of the results of traditional models under various preferred banding methods

The original spectral data obtained from the hyperspectral imaging system is 960*960*360, which contains 360 band numbers in the 380-1025nm band range, indicating that each sample data has 360 spectral reflection values. However, not all bands contribute to extracting meaningful features from the target. Most of the information obtained from most of these bands is essentially used for information. It is essential to select spectral feature wavelengths that carry useful information to address this issue. Three band optimization methods, including Sequential Projection Algorithm (SPA), Competitive Adaptive Reweighted Sampling (CARS), and Variable Iterative Space Shrinkage Approach (VISSA), were employed on the original spectral dataset. Collectively, these methodologies serve to extract pertinent information from the high-dimensional spectral data, effectively eliminating redundancy and irrelevant features.

In the experiment, the SPA algorithm identified 25 distinctive spectral bands, the CARS algorithm highlighted 68 relevant bands, and the VISSA algorithm pinpointed 123 significant bands. By employing band selection strategies, redundant information is significantly reduced, which is advantageous for model training and reduces computational time. Subsequently, the selected feature wavelengths were employed to construct the models for discerning the sweet corn seed viability. The experimental results are shown in **Table 4**.

**Table 4:** Average classification accuracy of the model under different band preferences.

Model	Band preference method					
	SPA		CARS		VISSA	
	Training accuracy (%)	Test accuracy (%)	Training accuracy (%)	Test accuracy (%)	Training accuracy (%)	Test accuracy (%)
SVM	88.91	86.52	89.33	86.89	89.62	**87.51**
ELM	89.29	87.26	90.08	88.55	89.87	**88.94**
1DLSTM	87.02	85.61	87.65	85.96	89.21	**86.47**
1DCNN	90.05	87.95	90.17	88.87	90.30	**89.06**

The bold font indicates that the algorithm has the best results compared to the other parties.

Firstly, when comparing the three band selection methods, VISSA demonstrated the best performance in the test set, followed by CARS, while SPA showed the poorest performance. This could be attributed to VISSA retaining the highest number of bands, which is favorable for model training. However, in terms of actual classification accuracy, the three band selection methods did not exhibit a significant difference, with less than a 2% gap between them. Secondly, 1DCNN still maintains the highest classification accuracy across different preprocessing methods, reaching 89.06%, followed by ELM. 1DLSTM performs the worst, with the SVM slightly outperforming 1DLSTM. The maximum difference in classification accuracy between the various models is around 3%. Finally, compared to the performance of various models across all spectral bands, there is a noticeable decrease in the performance of all algorithms, with classification accuracy decreasing by approximately 2% to 4% on average. Overall, the band selection strategy reduces the training time of the models to some extent, but it may also result in the exclusion of some valuable spectral information, thus leading to a decline in model performance.

### 3.4 Comparison of the effects of different deep neural networks

To facilitate the training of deep learning models, the spectral image data, initially containing 360 spectral bands, underwent dimensionality reduction using the Principal Component Analysis (PCA) algorithm. Following PCA treatment, a set of five spectral bands of images, each with a size of 46×46 pixels, within the 388-1025nm range, were employed to create various deep learning network-based spectral models. These models are proficient in effectively detecting different levels of vitality in sweet corn seeds. Subsequently, the CNN, LSTM, CNN-LSTM, and FA-CNN-LSTM models were applied to assess the classification accuracy. The training set and the test set were divided into an 7:3 ratio. The experimental results, hyperparameters for each deep learning model, and the optimizer selections are detailed in **Table 5**. The provided hyperparameters include batch size (bs) and learning rate (lr). Besides, all training and validation experiments are conducted on a deep learning workstation performed on an Ubuntu 20.04 LTS system with an Intel Xeon Gold 6130 CPU@2.1GHz 32 processors, two CUDA devices with dual Nvidia GeForce RTX 3090 GPUs, and 64 GB memory. The software is implemented on the PyTorch 1.13.0 framework and the PyTorch Image Models library with Python 3.9.

**Table 5:** Deep neural network recognition model based on 2D spectral image data.

Deep learning model	Parameters		Optimizer	Training accuracy (%)	Test accuracy (%)
	bs	lr			
CNN	1	0.001	SGD	97.17	94.59
LSTM	4	0.001	Adam	98.65	95.27
CNN-LSTM	2	0.001	SGD	98.82	95.94
FA-CNN-LSTM	2	0.002	SGD	**100**	**97.23**

The bold font indicates that the algorithm has the best results compared to the other parties.

It is worth noting that, in the training dataset, the classification accuracy of all models is nearly 100%. In particular, the proposed model achieves a classification accuracy of 100% on the training set. However, in terms of accuracy on the test set, the FA-CNN-LSTM model displayed the most impressive classification performance among all models, achieving the highest accuracy at 97.23%. Following closely were CNN-LSTM and LSTM, with CNN performing the least effectively, achieving an accuracy of 94.59%. Moreover, the performance of the FA-CNN-LSTM model significantly outperforms the other three deep learning models, with an approximately 2% higher accuracy on the test set. This notable improvement can be attributed to the FA algorithm’s superior optimization of the model’s hyperparameters, specifically batch size (bs) and learning rate (lr). When compared to traditional machine learning models, the deep learning models performed notably better. In particular, the accuracy of the FA-CNN-LSTM model on the test set is 5% higher than that of the 1DCNN model.

**Figure 11:**
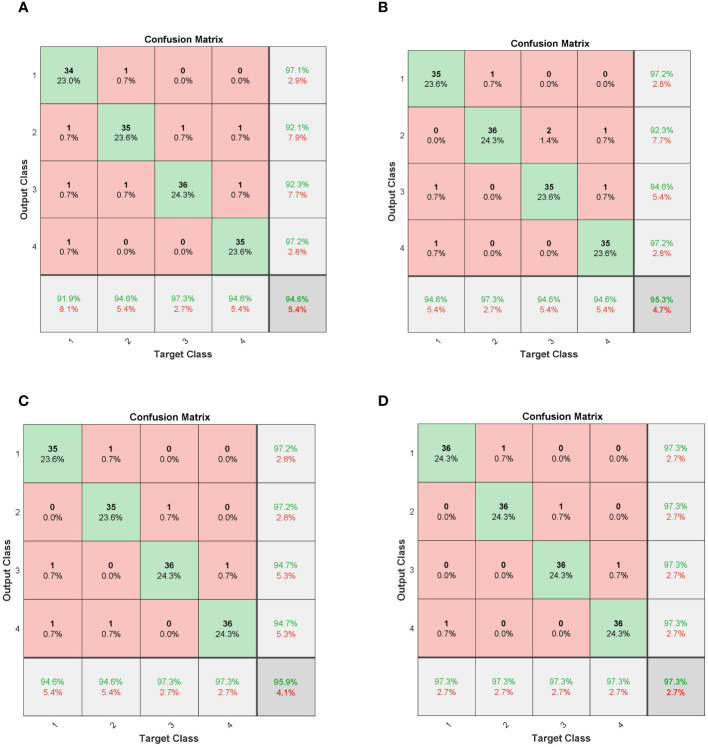
Confusion matrices for deep learning models. **(A)** CNN; **(B)** LSTM; **(C)** CNN-LSTM; **(D)** FA-CNN-LSTM.

To better analyze the experimental results from a visual perspective, **Figure 11** displays the confusion matrices of various neural network models in seed vitality detection. The relevant results are consistent with the data statistically summarized in **Table 5**, thereby effectively demonstrating the accuracy of the experimental results.

**Figure 12:**
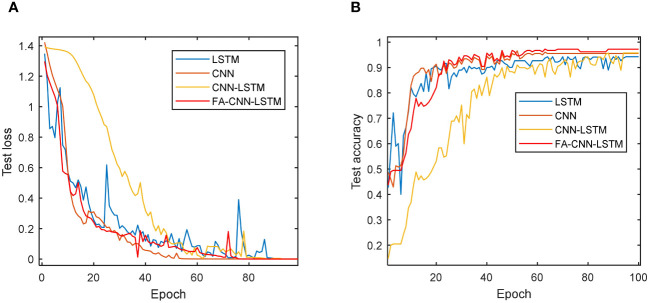
Loss function and accuracy curve of different models in 100 epochs: **(A)** Testing loss of four models; **(B)** Testing accuracy of four models.

Furthermore, **Figure 12** illustrates the accuracy and loss function evolution curves of four deep learning models on a test set. The loss function on the test set is constantly decreasing and almost approaching 0 for all models. CNN and FA-CNN-LSTM remained stable at 70 epochs. Similarly, both CNN and FA-CNN-LSTM models exhibit a stable accuracy trend after 70 epochs. More importantly, all models achieve a stable testing accuracy trend after nearly 100 epochs and reach their peak performance on the test data. The preceding experimental findings underscore the impressive capacity of deep learning models in discerning various vitality levels among sweet corn seeds. Additionally, these results emphasize the effectiveness of optimizing the CNN-LSTM model using the FA to enhance its overall performance.

## 4 Conclusion

Nondestructive seed vitality identification is beneficial for selecting high-quality seeds. This paper proposed hyperspectral imaging and deep learning model optimized by firefly algorithm to identify different viability sweet corn seeds. First, a total of 496 sweet corn seeds hyperspectral data of four different aging grades were collected. Then, traditional machine learning algorithms and deep learning methods are both utilized to construct seed vitality identification models, employing one-dimensional spectral data and spectral images, respectively. The experimental results indicated that the classification accuracy of traditional machine learning algorithms is significantly better across the full band compared to the selected bands. In comparison to traditional machine learning algorithms, the deep learning model exhibited a significant improvement in classification performance, achieving an accuracy rate exceeding 94.59%. In particular, the FA-CNN-LSTM had the best performance at 97.23%. Furthermore, the CNN-LSTM model optimized by the FA obtains superior parameter configurations, resulting in a 1.29% improvement in classification accuracy compared to the CNN-LSTM. In summary, it is worth noting that this approach demands a more substantial volume of image data compared to conventional machine learning algorithms. In future research, we plan to explore additional deep-learning models specifically designed for scenarios with limited sample data to improve the identification of sweet corn seed varieties.

## Data availability statement

The original contributions presented in the study are included in the article/supplementary material. Further inquiries can be directed to the corresponding author.

## Author contributions

YW: Conceptualization, Data curation, Writing – original draft. SS: Supervision, Writing – review & editing.
